# Real-World Insights Into Dementia Diagnosis Trajectory and Clinical Practice Patterns Unveiled by Natural Language Processing: Development and Usability Study

**DOI:** 10.2196/65221

**Published:** 2025-02-25

**Authors:** Hunki Paek, Richard H Fortinsky, Kyeryoung Lee, Liang-Chin Huang, Yazeed S Maghaydah, George A Kuchel, Xiaoyan Wang

**Affiliations:** 1 IMO Health, Rosemont, IL, United States; 2 UConn Center on Aging, University of Connecticut School of Medicine, Farmington, CT, United States; 3 Center for Quantitative Medicine, University of Connecticut School of Medicine, 195 Farmington Ave, Farmington, CT, 06032, United States, 1 201-282-8098; 4 Department of Health Policy and Management, Tulane University, New Orleans, LA, United States

**Keywords:** dementia, memory loss, memory, cognitive, Alzheimer disease, natural language processing, NLP, deep learning, machine learning, real-world insights, electronic health records, EHR, cohort, diagnosis, diagnostic, trajectory, pattern, prognosis, geriatric, older adults, aging

## Abstract

**Background:**

Understanding the dementia disease trajectory and clinical practice patterns in outpatient settings is vital for effective management. Knowledge about the path from initial memory loss complaints to dementia diagnosis remains limited.

**Objective:**

This study aims to (1) determine the time intervals between initial memory loss complaints and dementia diagnosis in outpatient care, (2) assess the proportion of patients receiving cognition-enhancing medication prior to dementia diagnosis, and (3) identify patient and provider characteristics that influence the time between memory complaints and diagnosis and the prescription of cognition-enhancing medication.

**Methods:**

This retrospective cohort study used a large outpatient electronic health record (EHR) database from the University of Connecticut Health Center, covering 2010‐2018, with a cohort of 581 outpatients. We used a customized deep learning–based natural language processing (NLP) pipeline to extract clinical information from EHR data, focusing on cognition-related symptoms, primary caregiver relation, and medication usage. We applied descriptive statistics, linear, and logistic regression for analysis.

**Results:**

The NLP pipeline showed precision, recall, and *F*_1_-scores of 0.97, 0.93, and 0.95, respectively. The median time from the first memory loss complaint to dementia diagnosis was 342 (IQR 200-675) days. Factors such as the location of initial complaints and diagnosis and primary caregiver relationships significantly affected this interval. Around 25.1% (146/581) of patients were prescribed cognition-enhancing medication before diagnosis, with the number of complaints influencing medication usage.

**Conclusions:**

Our NLP-guided analysis provided insights into the clinical pathways from memory complaints to dementia diagnosis and medication practices, which can enhance patient care and decision-making in outpatient settings.

## Introduction

The rising prevalence of dementia, driven by an aging population, presents a profound concern for society [1-4], placing substantial burdens on individuals and imposing high financial costs [5-7]. Despite these challenges, no curative treatments are currently available [8,9], highlighting the critical need for early detection of prodromal symptoms of dementia, such as mild cognitive decline [10-13], and timely diagnosis. Early intervention can delay disease progression or alter the trajectory toward dementia [9,14,15]. However, dementia and its associated symptoms are frequently underreported and underdiagnosed in clinical practice [16,17]. As the condition progresses, patients commonly experience increased memory loss, deteriorating cognitive ability, heightened confusion, and changes in personality like agitation. The conversion from mild cognitive impairment to Alzheimers disease has been explored using patient health data [18].

Electronic health records (EHRs) offer a valuable resource for enhancing the detection and management of disease by providing comprehensive data on patient health and history [19-23]. However, much of the nuanced patient information is embedded within unstructured clinical notes and is not accessible through structured data. Natural language processing (NLP), a subfield of artificial intelligence that enables computers to understand, interpret, and generate human language, holds promise for extracting meaningful information from vast and complex free-text EHRs [24-27]. NLP has been instrumental in automatically extracting clinical information in various medical domains, including geriatric care [28-35]. For instance, Kharrazi et al [36] showcased higher rates of geriatric syndrome extraction from unstructured EHR using NLP compared to relying solely on claim data or structured EHR data. Studies have successfully extracted cognitive status and measurement scores [37,38], as well as lifestyle exposures and discourse production for Alzheimers disease [39], from clinical documentation using NLP. Additionally, multiple studies have applied NLP methods to extract neuropsychiatric symptoms and cognitive or function impairment information [40-43]. State-of-the-art models, such as pretrained Bidirectional Encoder Representation from Transformer (BERT), have been applied in clinical settings for tasks like detecting inpatient falls [44] and classifying dementia risk [45] using clinical notes. While previous research has made significant strides in the earlier detection of cognitive decline using EHR, most studies have focused on extracting symptoms or cognitive measurement scores rather than other clinical features that affect disease progression, such as the relationship of the primary caregivers with patients.

We aimed to investigate the time interval from initial memory loss complaints to dementia diagnosis and explore the association between various clinical features, including the family primary caregiver relationship, using real-world outpatient clinical notes. Additionally, we aimed to analyze the pattern of cognition-enhancing medication prescriptions before diagnosis. To achieve this, we developed a customized NLP pipeline using deep learning techniques, based on a prodromal dementia symptom ontology that we established.

## Methods

### Study Cohorts

**Figure 1.**
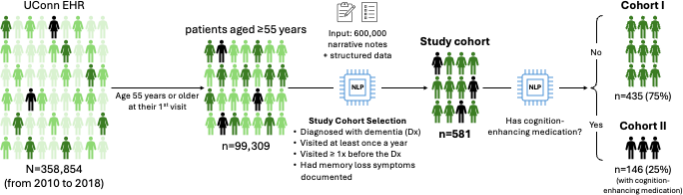
The workflow for the study cohort selection and information extraction. EHR: electronic health record; NLP: natural language processing.

This retrospective study used data from the UConn Health Center between 2010 and 2018. The use of longitudinal EHR data allowed us to track all patients’ clinical information, including demographic characteristics, diagnoses, measurements, medications, and signs. The study cohort was defined as patients who met the following criteria: (1) received a dementia diagnosis, (2) had at least one outpatient visit per year, (3) had at least one visit before the dementia diagnosis, and (4) had documented memory loss–related symptoms (eg, memory loss, confusion, cognition impairment, trouble remembering, not recalling, forgetting, and blackout) in the EHR. Dementia was defined based on the presence of 3 or more *ICD* (*International Classification of Diseases*) codes used for dementia phenotyping in our study (as detailed in Multimedia Appendix 1) and dementia documentation in their clinical notes. We analyzed demographic and clinical characteristics from structured EHR data, including insurance details, the initial location (medical unit) of memory loss complaints, and the location of the first dementia diagnosis. These locations encompass various settings within this health care system, such as geriatric medicine, internal medicine, and neurology outpatient clinics. NLP was used to extract symptoms and primary caregiver (family supporter) relationship information from clinical notes. Both diagnosis and cognition-improving medication information were extracted from both structured and unstructured data. Figure 1 provides an overview of the selection process of the study cohort and the information extraction process.

### Building a Deep Learning–Based NLP Pipeline

A framework was developed to curate the prodromal dementia symptoms comprising four stages: (1) preprocessing and query expansion, (2) ontology construction and annotation, (3) NLP model development, and (4) system evaluation.

#### Preprocessing and Query Expansion

In this stage, a query was expanded to extract and identify a broad set of patient notes with documented memory loss symptoms. A list of seed terms was obtained through a manual survey of the literature and a clinical note review by a clinical researcher and domain expert. A bigram word2vec algorithm [46] was used to identify additional significant terms potentially related to memory loss symptoms to ensure the encapsulation of an expansive cohort. The expanded, rule-based query terms provided in Multimedia Appendix 2 were subsequently applied to extract the relevant patient notes for NLP modeling.

#### Ontology Construction and Annotation

**Figure 2.**
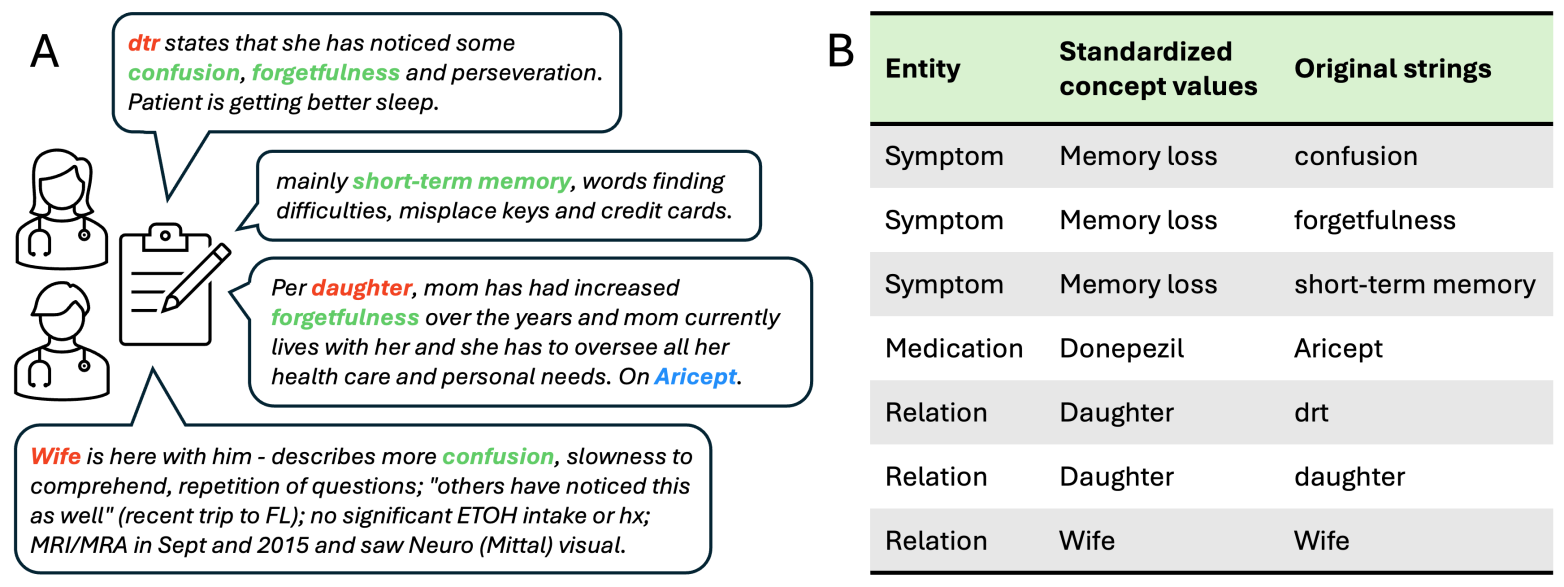
The ontology of memory loss and the annotated sample note. (A) The ontology of prodromal dementia symptoms. In total, 9 entities and 9 semantic relations between entities were defined in the ontology. (B) A sample note that has been deidentified and annotated with prodromal dementia symptoms. In this note, the following entities have been annotated and related to each other: symptoms, relation, medication, duration, status change, test, and temporal. MRI/MRA: magnetic resonance imaging/magnetic resonance angiography, dtr:daughter, ETOH: ethanol, FL: florida, hx: history.

This stage involves the simulation of an expert’s knowledge and understanding of the free text. A prodromal dementia symptom ontology was built based on the physician’s opinion, a comprehensive literature review, and a clinical note review. The ontology includes 9 entities and 9 relations. The 9 entities are “memory loss symptom (Sx),” “dementia diagnosis (Dx),” “temporal,” “duration,” “status change: worse,” “other symptoms,” “cognitive test result,” “caregiver relation,” and “cognition enhancing medication (Rx).” The 9 relations are “has complaint date,” “has diagnosis date,” “has other symptom information,” “has status change information,” “has duration information,” “has test information,” “has caregiver information,” “has treatment information,” and “has effects” as depicted in Figure 2A. Two independent annotators manually annotated notes using Clinical Language Annotation, Modeling, and Processing (CLAMP) [47], an NLP toolkit, guided by the constructed ontology. Figure 2B shows an example note with entities and relations annotated.

#### NLP Model Development

The annotated notes (n=815) obtained in the previous stage were used for NLP model training. These notes were randomly split into a training set (80%, n=652, of annotated notes) and an independent validation set (the remaining 20%, n=163). The manual annotation and training processes were iteratively performed with additional manually annotated notes to enhance model performance until the model achieved an *F*_1_-score of >0.8. For model training, a multilayer deep learning architecture was adopted, which involved transforming the text into sequential vectors of characterization through the embedding step. The vectors were then fed into a Bidirectional Long Short-Term Memory (BiLSTM), a text classification architecture based on artificial neural networks, for pattern recognition in both forward and backward directions. Finally, the patterns were sent to the next layer of a Conditional Random Field (CRF) model for prediction probability computation. BiLSTM-CRF architectures are widely used in clinical NLP tasks, demonstrating robustness in recognizing entities in sequential data like clinical notes [48] and effectively leveraging moderate-sized datasets with lower computational resource requirements.

#### NLP Pipeline Evaluation

The performance of the pipeline was evaluated in the validation set through precision (positive predictive value [PPV]), recall (sensitivity), and *F*_1_-score (a balanced score between false positives [FPs] and false negatives [FNs]). Recall was calculated as the ratio of the number of entities that were identified by the pipeline over the total number of the corresponding entities in the manually annotated gold standard (ie, true positive [TP]/[TP+FN]). Precision was measured as the ratio of the number of distinct entities returned by the correct pipeline according to the gold standard divided by the total number of entities found by our pipeline (ie, TP/[TP+FP]). *F*_1_-score was calculated as the harmonic mean of PPV and sensitivity (ie, 2 × PPV × sensitivity / [PPV + sensitivity]).

### Standardization of Concept Values Leveraging NLP

**Figure 3.**
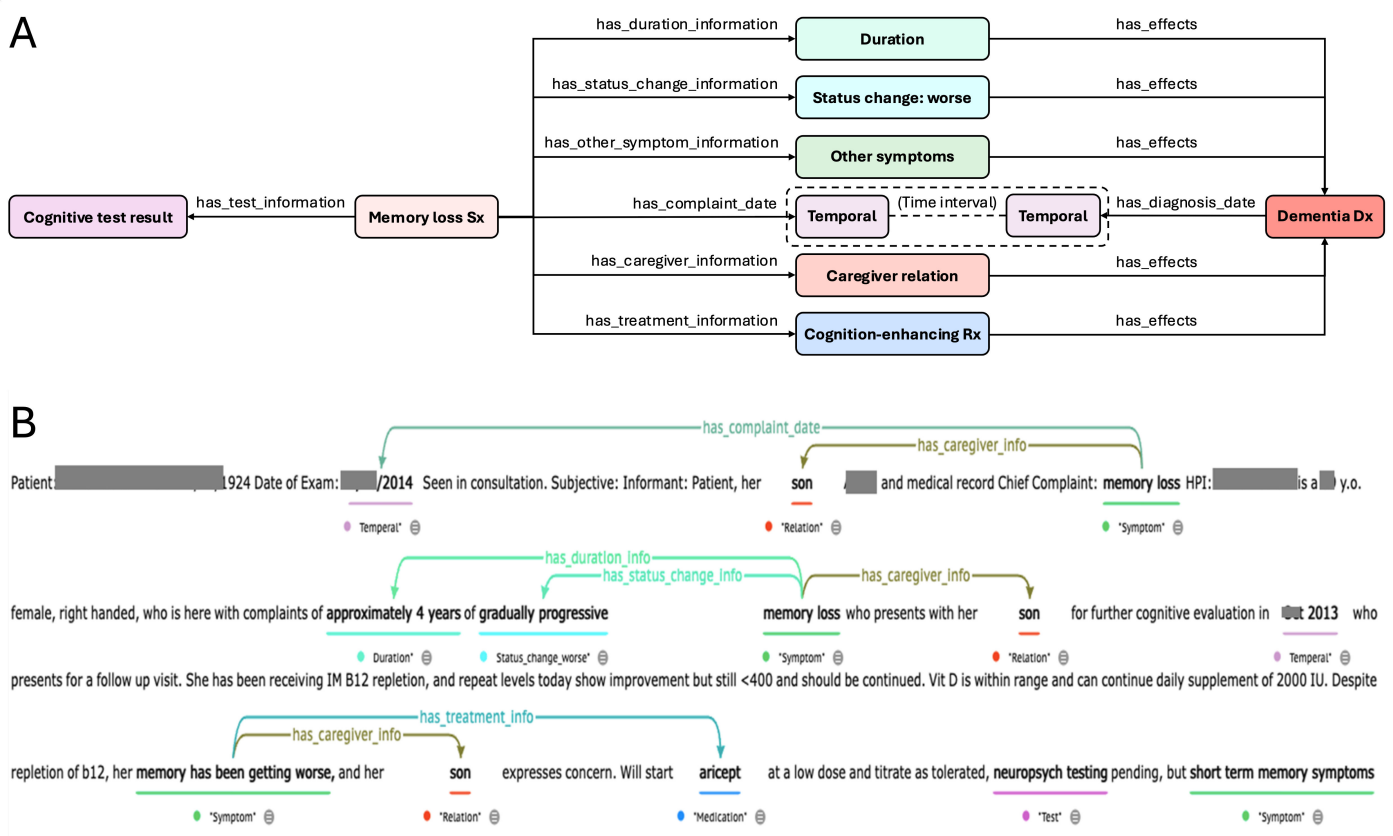
Clinical note standardization via NLP. (A) The diverse expressions used in clinical notes depict concepts such as memory loss symptoms, primary caregiver relationships, and cognition-enhancing medications. (B) A sample of the standardized output generated by the NLP process, representing the standardized values of the various original expressions. Dx: diagnosis; HPI: history of present illness; NLP: natural language processing; Rx: medication; Sx: symptom.

Clinical notes contain abundant information but are often heterogeneous in form. To enable use case analysis, these heterogeneous entities needed to be standardized. Figure 3A illustrates the various forms in which cognition-related concepts like forgetfulness, memory loss, short-term memory, and confusion were documented in the clinical notes. Abbreviations (eg, dtr for daughter) and mixed use of brand names and generic names of the same drugs (eg, Aricept and donepezil) were commonly found. Figure 3B provides examples of standardized concept values obtained through the NLP process. The cognition-enhancing medications discussed include donepezil, memantine, rivastigmine, and galantamine. For the analysis, the son, son-in-law, daughter-in-law, grandson, and granddaughter were classified as other adult children, while the nephew, niece, cousin, brother, and sister were classified as other family support.

### Characterization of Dementia Cohorts and Longitudinal Analysis of Dementia Trajectory

The collection of clinical information of patients was accomplished through the extraction of both structured and unstructured EHR data. The statistical analysis was performed using R software (R Core Team). A logistic regression model was used to assess the relationship between medication prescription history and other factors, such as memory loss complaints and the primary caregiver relation information, by calculating the odds ratio (OR) and 95% CI. A total of 149 patients who had memory loss complaints and diagnoses recorded on the same day were excluded from this study.

### Ethical Considerations

This study involved no interaction with patients as it is a retrospective cohort study that used a deidentified dataset. As such, it was deemed as exempt from institutional review board approval.

## Results

### Building a Memory Loss NLP Pipeline

The performance of our memory loss NLP pipeline was evaluated using precision, recall (sensitivity), and *F*_1_-score metrics in the validation set, with detailed results presented in Multimedia Appendix 3. Our system achieved high scores across all semantic types, including “memory loss symptom,” “dementia diagnosis,” “duration,” “primary caregiver,” and “status change” (overall precision, recall [sensitivity], and *F*_1_-scores of 0.97, 0.93, and 0.95, respectively). For example, the precision for “memory loss symptoms” was 0.97, indicating that 97% of “memory loss symptoms” were identified by our NLP system in patients’ clinical notes, as verified against a manually curated gold standard. The recall (sensitivity) of 0.93 implies that our system correctly identified 93% of actual memory loss cases, with only 7% missed. Multimedia Appendix 3 provides comprehensive details for various semantic types and relations, enabling a thorough understanding of the NLP pipeline’s performance.

### Clinical Characterization of Study Cohorts

#### Cohort Identification

An average of 358,854 patients visited the UConn Health system for outpatient care between 2010 and 2018, with the number of visits increasing annually from 224,488 in 2010 to 1,024,349 in 2017. There were 8686 patient visits in 2018 until the time point we collected data. Out of these patients, 99,039 patients were aged 55 years or older at their first visit. From more than 600,000 narrative records and coded data of those patients, we identified 730 patients who had at least one outpatient visit per year, at least one visit before their dementia diagnosis, and documented memory loss symptoms in their clinical notes. Of these, 149 (20.4%) patients reported memory loss complaints and were diagnosed with dementia on the same day, while 581 (79.6%) patients had at least a 1-day gap between the complaint and diagnosis. For the following analysis, 581 patients with memory loss symptoms documented at different days were included in study cohorts (Figure 1).

#### Cohort Demographics

The demographic characteristics, primary insurance, and medication information of study cohorts are summarized in Table 1. Most cohort members were non-Hispanic White individuals (509/581, 87.6%) and female (381/581, 65.6%), with an age distribution of over 85 years (315/581, 54.2%), 75‐84 years (171/581, 29.4%), 65-74 years (63/581, 10.8%), and under 65 years (32/581, 5.5%).

**Table 1.** Demographic characteristics, primary insurance, and medication information of study cohorts (N=581).

Characteristics		Values, n (%)
Age (years)		
	<65	32 (5.5)
	65‐74	63 (10.8)
	75‐84	171 (29.4)
	85+	315 (54.2)
Race		
	White	509 (87.6)
	African American	33 (5.7)
	Others	39 (6.7)
Sex		
	Female	381 (65.6)
	Male	200 (34.4)
Primary caregiver (family supporter) relation (n=291)		
	Husband	26 (8.9)
	Wife	39 (13.4)
	Daughter	135 (46.4)
	Son	50 (17.2)
	Other family members	41 (14.1)
	Missing	290 (49.9)
Prior medication before the first diagnosis of dementia		146 (25.1)

#### Primary Caregiver and Medication Information

Out of 291 patients with primary caregiver relation information, adult children were the main caregivers (n=185, 63.6%, with n=135, 46.4%, being daughters and n=50, 17.2%, being sons), followed by spouses (n=65, 22.3%, with n=39, 13.4%, being wives and n=26, 8.9%, being husbands). Other family members (eg, nephew) made up 14.1% (41/291) of the cohort. Out of 581 patients, 146 (25.1%) patients had been prescribed cognition-improving medications prior to the dementia diagnosis.

#### Outpatient Care Locations

Next, we investigated the outpatient care locations where the first memory loss complaints were reported and where dementia was diagnosed. Geriatrics is the most frequent location for both the first memory loss complaints made (308/581, 53%) and the diagnosis of dementia (350/581, 60.2%), followed by primary care (185/581, 31.8%, and 163/581, 28.1%, respectively) and neurology (39/581, 6.7%, and 61/581, 10.5%, respectively). The majority of the cohort was covered by Medicare (354/581, 60.9%) or Medicaid (72/581, 12.4%) as primary insurance, while 26.2% (152/581) had commercial insurance. Only a small percentage of patients (2/581, 0.3%) had no insurance coverage (Multimedia Appendix 4).

### Distribution of Time Intervals Between Cognitive Symptom Complaints and Dementia Diagnosis and the Number of Complaints

#### Time Interval

The median time interval between the first memory loss complaints and dementia diagnosis was 342 days, ranging from a minimum of 1 day to a maximum of 1458 days in our study cohort (n=581) (Multimedia Appendix 5).

#### Health Care Use

Additionally, the number of complaints made before being diagnosed was analyzed, with a median of 3 complaints, ranging from a minimum of 1 complaint to a maximum of 18 complaints (Multimedia Appendix 5).

### Association Analysis for the Earlier Dementia Diagnosis

We aimed to identify the clinical features that are associated with earlier diagnosis of dementia from the first memory loss complaints. Results indicated that the location of the first complaints made and the diagnosis, as well as the relation of the primary caregiver, were significantly associated with earlier diagnosis of dementia. Patients who made complaints in geriatrics (−141 days, *P*<.001, *χ*² test) or neurology (−158 days, *P=*.02) were diagnosed with dementia earlier compared to those who made complaints in primary care. Furthermore, patients diagnosed with dementia in geriatrics had a shorter interval of 152.9 days (*P*<.001) compared to those diagnosed in primary care. Additionally, having a wife or a daughter as a primary caregiver was associated with an earlier diagnosis of dementia, with a shorter interval of 249.6 days (*P=*.01) and 176.8 days (*P=*.04), respectively, compared to those who had a husband as a primary caregiver. However, factors such as age or insurance types were not found to have a significant impact on earlier diagnosis (Table 2).

**Table 2.** Statistical analysis of time intervals between the first complaints and dementia diagnosis.

Features		Estimate (95% CI)	*P* value (*χ*² test)
Age (years)			
	<65	109.8 (–26.7 to 246.3)	.12
	65‐74	–61.6 (–163.2 to 40)	.24
	75‐84	0.08 (–69.4 to 69.6)	≥.99
	85+	Ref^a^	—^b^
Primary insurance			
	No insurance	36.8 (–25 to 98.6)	.24
	Medicaid	5.8 (–255.5 to 267.1)	.97
	Medicare	36.8 (–11.9 to 85.5)	.14
	Commercial	Ref	—
The location of the first memory loss complaint			
	Geriatrics	–141 (–212 to –70)	<.001
	Neurology	–158 (–286.2 to –29.8)	.02
	Primary care	Ref	—
	Other	81 (–35.1 to 197.1)	.17
The location of the first diagnosis of dementia			
	Geriatrics	–152.9 (–244 to –61.8)	<.001
	Neurology	–82.2 (–191.4 to 27)	.14
	Primary care	Ref	—
	Other	42 (–225.4 to 309.4)	.76
Primary caregiver (family supporter) relation			
	Wife	–249.6 (–437.2 to –62)	.009
	Daughter	–176.8 (–346.1 to –7.5)	.04
	Other adult children	–127.4 (–256.9 to 2.1)	.05
	Other family support	–257.5 (–577.1 to 62.1)	.11
	Husband	Ref	—

a Reference.

b Not applicable.

### Association Analysis for the Medication Usage

Medication was prescribed in 25.1% (146/581) of patients before dementia diagnosis. We next analyzed factors associated with the usage of cognition-enhancing medication before the diagnosis of dementia after the 1st complaints of memory loss. The only factor that was significantly associated with medication usage was the total number of memory loss complaints made; each additional memory complaint was associated with a 15% greater likelihood that cognition-enhancing medications were prescribed (OR 1.148, 95% CI 1.027‐1.283; Table 3).

**Table 3.** An analysis of the factors associated with the usage of medication before the diagnosis of dementia.

Features		OR^a^ (95% CI)
Age (years)		
	<65	3.827 (0.403‐23.32)
	65‐74	1.251 (0.507‐3.727)
	75‐84	1.127 (0.615‐2.445)
	85+	Ref^b^
The location of the first memory loss complaint		
	Geriatrics	1.477 (0.551‐3.959)
	Neurology	2.124 (0.449‐10.05)
	Primary care	Ref
	Other	0.331 (0.103‐1.058)
The location of the first diagnosis		
	Geriatrics	0.489 (0.172‐1.39)
	Neurology	0.65 (0.182‐2.319)
	Primary care	Ref
Family support		
	Wife	4.367 (0.85‐22.447)
	Daughter	1.831 (0.263‐12.74)
	Other adult children	1.609 (0.538‐4.816)
	Other family support	1.033 (0.276‐3.871)
	Husband	Ref
Total number of memory loss complaints before the diagnosis of dementia		1.148 (1.027‐1.283)

a OR: odds ratio.

b Reference.

## Discussion

### Principal Findings

We developed a high-performance deep learning–based NLP algorithm on an EHR dataset of dementia patients to delve into the real-world trajectory of dementia, starting from initial memory loss complaints to dementia diagnosis. Our investigation focused on the time interval from the first memory loss complaints to dementia diagnosis, the proportion of prescribed cognition-enhancing medication before diagnosis during this trajectory, and the clinical characteristics associated with these features.

We found that 20.4% (149/730) of patients had same-day documentation of memory loss complaints and dementia diagnosis. Among the remaining 79.5% (580/730) of patients with at least a 1-day gap between complaints and diagnosis, over half of the patients received a dementia diagnosis within a year of their initial memory loss complaints, with a median time of 342 days. The location of the first complaint and diagnosis and the relationship with the primary caregiver emerged as influential factors in achieving an earlier diagnosis. Notably, patients who initiated complaints or were diagnosed in geriatrics or neurology received earlier diagnoses compared to those in primary care. This underscores the important role of the initial complaint’s location and the dementia diagnosis’s setting in the early detection and management of dementia. Our findings align with previous research indicating missed and delayed diagnoses in primary care [49]. Geriatricians and neurologists possess significantly more expertise and practical experience in diagnosing dementia and prescribing these meds than most primary care doctors. To enhance early detection in primary care, it is crucial to train primary care providers to recognize the nuances of dementia symptoms and to appreciate the importance of thorough assessments during initial consultations. This approach could significantly reduce delays in diagnosis. Furthermore, improving caregiver education regarding the signs and symptoms of dementia is essential, as it can lead to earlier recognition of concerns and encourage timely visits to health care professionals. Additionally, understanding the factors that lead patients to receive care in a geriatric or neurological department rather than primary care would be an important question for further investigation. Similar to the previous study that identified dementia severity and marital status as independent predictors of receiving a clinical cognitive evaluation [50], other factors such as more complex medical needs (eg, multiple chronic conditions and polypharmacy), severe function decline, and the primary caregiver’s educational level or relationship with the patient could be associated with visits to geriatric or neurologic departments.

Remarkably, we found that patients with a wife or daughter as their primary caregiver were diagnosed earlier and more frequently used cognitive-improving medication before the dementia diagnosis. This emphasizes the vital role of primary caregivers in the diagnosis and treatment of dementia patients. Mahmoudi et al [35] previously emphasized the importance of extracting caregiver information in dementia patient notes and developed the rule-based NLP algorithm to identify caregiver availability. In our work, we extended this by also extracting family-caregiver relationships with patients and analyzing their impact on the early diagnosis of dementia. Subsequent research should explore the underlying mechanisms and factors of caregivers in this context such as the association between the relationship of primary caregivers and visits to geriatric or neurologic departments. Contrary to expectations, our study revealed that age and insurance were not associated with earlier diagnoses.

Surprisingly, the total number of memory loss complaints emerged as the sole factor significantly linked to medication usage, with other factors showing no significant association. The correlation between increased medication prescriptions and additional memory loss complaints highlights health care providers’ responsiveness to escalating symptoms and underscores the importance of proactive monitoring of cognitive symptoms. This finding aligns with the Alzheimer’s Association guidelines for early identification and treatment of Alzheimers disease, particularly in its initial stages. By recognizing and documenting memory loss complaints, clinicians can better initiate appropriate therapeutic interventions, particularly those aimed at altering disease progression. Additionally, our findings emphasize the need for enhanced caregiver education on the importance of reporting memory loss and other cognitive changes.

We demonstrated that extracting cognitive symptom-related terms from longitudinally documented patient notes before dementia diagnosis could be an alternative approach to analyzing documented cognitive measurement scores during patient visits, potentially aiding in identifying dementia patients. Previous studies have highlighted a significant lack of such documentation in clinical notes [37,41,51]. For instance, Harding et al [51] found that cognitive measurement scores were rarely available in their cohorts when establishing the algorithm for identifying dementia patients in EHR. Similarly, Maserejian et al [37] demonstrated a low percentage of dementia (11%) or Alzheimers disease (24%) in patients with cognitive measurement scores such as Mini Mental State Examination (MMSE), a recall test, a clock drawing, Montreal Cognitive Assessment (MoCA), Mini-Cog, or Saint Louis University Mental Status (SLUMS) documented and suggested prompts of cognitive measurement. McCoy et al [41] attempted to extract cognitive symptom-related terms (eg, impulsive, forgetful, cognitive, and memory) and converted them into scores, given the issue of reliability and scalability of the cognitive measurement test. Consistently, the proportion of patient notes with cognitive test names, including MMSE, SLUMS, MoCA, Mini-Cog, clock drawing, trail making, Boston naming test, and Wisconsin card sorting test, was very low in our study, so these were not used in further analysis.

Our NLP approach in automatically identifying cognitive symptom-related terms and primary caregivers, and systematically analyzing these factors along with other structured data, enhanced our understanding of dementia progression and management. This approach provides a practical and scalable method for identifying cognitive impairment, especially when traditional cognitive measurement scores are lacking. Further exploration using this NLP method could significantly advance the field, providing deeper insights and more effective interventions for dementia care. Moreover, our study findings align with the Alzheimer’s Association’s recommendations for using simple practical tests like Mini-Cog or General Practitioner Assessment of Cognition (GPCOG), developed by a group of clinical dementia experts, during annual visits, particularly when symptoms are reported by patients or caregivers, thereby supporting current clinical practice in dementia care.

### Strengths and Limitations

Our study has several strengths. First, our work presents a novel approach to understanding clinical practices in dementia by examining the time interval from the symptom complaints to the diagnosis of dementia using real-world data. Second, our study highlights how the relationship between primary caregivers and patients and the location (medical units: geriatrics, neurology, and primary care) of complaints made may influence the time to diagnosis. While existing ontologies provide comprehensive medical concepts, they often lack the specificity necessary to accurately capture the relationships between concepts. By defining the relationships between entities such as “has date” and “has caregiver information” in the ontology, our study could provide deeper insights into the clinical features that affect the time interval from the symptom presentation to dementia diagnosis in real-world contexts. Third, our study developed and validated a customized NLP model to be used to predict an outcome in a clinical setting using EHRs.

Several limitations of this study should be considered. First, the study relied on EHR data from a single health care system, which may limit the generalizability of the findings to other populations and health care settings. This system-specific reliance may introduce potential biases related to local clinical practices, documentation standards, and patient demographics, which could affect the study’s findings if applied to broader or more diverse health care environments. Additionally, the patient population in our dataset was predominantly White, female, and older patients above 85 years with Medicare or Medicaid insurance, which may further limit the generalizability of the findings to other demographic groups, including younger patients, males, and individuals from diverse racial backgrounds. Future studies should aim to include a more diverse patient population in terms of age, race, and insurance type across multiple health care systems to validate and potentially broaden the applicability of the findings.

Another limitation is the potential for loss of follow-up within the EHR data, as patients with less frequent or inconsistent visits may have different clinical trajectories. We preselected patients who had been diagnosed with dementia, had at least one documented memory loss complaint before diagnosis, and visited the health care system at least once per year to reduce the likelihood of significant follow-up loss. Nonetheless, variability in follow-up could still influence our results. Furthermore, potential biases within EHR documentation may impact the findings. For example, memory complaints may be underreported or inconsistently documented, depending on clinician practices and the completeness of note-taking. This variability could affect the accuracy of data extraction and the insights derived from the patient journey. Lastly, the study did not account for the potential impact of other medical conditions on the result. Incorporating objective measures of cognitive decline and other co-occurring neuropsychiatric symptoms could enhance the assessment of dementia.

### Conclusions

Our study highlights the importance of the location of initial memory loss complaints, the location of the dementia diagnosis, and the role of the primary caregiver in the early diagnosis and treatment of dementia patients. By analyzing complex clinical dementia care practice patterns within a real-world setting on a large scale using NLP, our exploratory analysis demonstrates the potential of advanced analytical techniques in achieving earlier and more accurate diagnoses of dementia.

## Supplementary material

10.2196/65221 Multimedia Appendix 1 *ICD* (*International Classification of Diseases*) codes used for the phenotyping of dementia cohort.

10.2196/65221 Multimedia Appendix 2 Query terms used for identifying memory loss–related symptoms.

10.2196/65221 Multimedia Appendix 3 Evaluation of memory loss NLP pipeline. NLP: natural language processing.

10.2196/65221 Multimedia Appendix 4 Descriptive statistics of providers and insurance information.

10.2196/65221 Multimedia Appendix 5 The distribution time intervals and complaints. (A) Distribution of the time intervals between the first memory loss complaints and the diagnosis of dementia. (B) Distribution of the number of complaints made before the diagnosis of dementia.

## Abbreviations

BERT: Bidirectional Encoder Representation from Transformer
BiLSTM: Bidirectional Long Short-Term Memory
CLAMP: Clinical Language Annotation, Modeling, and Processing
CRF: Conditional Random Field
EHR: electronic health record
FN: false negative
FP: false positive
GPCOG: General Practitioner Assessment of Cognition
*ICD*: *International Classification of Diseases*
MMSE: Mini Mental State Examination
MoCA: Montreal Cognitive Assessment
NLP: natural language processing
OR: odds ratio
PPV: positive predictive value
SLUMS: Saint Louis University Mental Status
TP: true positive
