# Supplementary material for: Real-World Insights Into Dementia Diagnosis Trajectory and Clinical Practice Patterns Unveiled by Natural Language Processing: Development and Usability Study
Source: JMIR Aging. 2025 Feb 25;8:e65221. doi: 10.2196/65221 (PMC11878476; doi:10.2196/65221)
Supplement: Multimedia Appendix 1 [file aging-v8-e65221-s001.docx]

## **Multimedia Appendix 1.** ICD codes used for the phenotyping of dementia cohort

| Codes | Description |
| --- | --- |
| A81.2 | (Dementia,) progressive multifocal leukoencephalopathy |
| F01.50 | Dementia, vascular without behavioral disturbance |
| F01.51 | Dementia, vascular with behavioral disturbance |
| F02.80 | Dementia in other diseases classified elsewhere without behavioral disturbance |
| F02.81 | Dementia in other diseases classified elsewhere with behavioral disturbance |
| F03.90 | Dementia, unspecified without behavioral disturbance |
| F03.91 | Dementia, unspecified with behavioral disturbance |
| F06.8 | (Dementia,) other specified mental disorders due to known physiological condition |
| G23.8 | (Dementia,) other specified degenerative diseases of the basal ganglia |
| G30.0 | (Dementia,) Alzheimer’s disease with early onset |
| G30.1 | (Dementia,) Alzheimer’s disease with late onset |
| G30.9 | (Dementia,) Alzheimer’s disease, unspecified |
| G31.1 | (Dementia,) senile degeneration of the brain, NOS |
| G31.9 | (Dementia,) Degenerative disease of the nervous system, unspecified |
| G31.01 | (Dementia,) Pick’s disease |
| G31.09 | (Dementia,) other frontotemporal dementia |
| G31.83 | (Dementia,) neurocognitive disorder with Lewy Bodies |
| G31.89 | (Dementia,) other specified degenerative diseases of the nervous system |
| G94 | (Dementia,) other disorders of the brain in diseases classified elsewhere |
