# Supplementary material for: Real-World Insights Into Dementia Diagnosis Trajectory and Clinical Practice Patterns Unveiled by Natural Language Processing: Development and Usability Study
Source: JMIR Aging. 2025 Feb 25;8:e65221. doi: 10.2196/65221 (PMC11878476; doi:10.2196/65221)
Supplement: Multimedia Appendix 2 [file aging-v8-e65221-s002.docx]

## **Multimedia Appendix 2.** Query terms used for identifying memory loss related symptoms

| **Query Terms** | \| forgets \| \| --- \| \| confusion \| \| memories loss \| \| forget \| \| dementia \| \| memory \| \| dementia, memory \| \| confusing \| \| mental \| \| forgetful \| \| remembering \| \| activity of daily living  ADLs  IADLs \| \| confused \| \| cognition \| \| doesn't recall \| \| trouble remembering \| \| confusion, memory \| \| mentally \| \| memory loss \| \| cognitive function \| \| forgetfulness \| \| Alzheimer's disease \| \| acute confusion \| \| forgets \| \| blackout \| \| mental status \| |
| --- | --- | --- | --- | --- | --- | --- | --- | --- | --- | --- | --- | --- | --- | --- | --- | --- | --- | --- | --- | --- | --- | --- | --- | --- | --- | --- | --- |
