# Supplementary material for: Real-World Insights Into Dementia Diagnosis Trajectory and Clinical Practice Patterns Unveiled by Natural Language Processing: Development and Usability Study
Source: JMIR Aging. 2025 Feb 25;8:e65221. doi: 10.2196/65221 (PMC11878476; doi:10.2196/65221)
Supplement: Multimedia Appendix 3 [file aging-v8-e65221-s003.docx]

## **Multimedia Appendix 3.** Evaluation of memory loss NLP pipeline.

| **Semantic** | **# of Entity** | **Entity** | | | **Relation** | | | |
| --- | --- | --- | --- | --- | --- | --- | --- | --- |
|  |  | **Precision** | **Recall** | **F-Score** | **Precision** | **Recall** | **F1** |  |
| Memory Loss Symptom | 4178 | 0.98 | 0.97 | 0.97 | 0.97 | 0.92 | 0.94 |  |
| Dementia Diagnosis | 873 | 0.99 | 0.96 | 0.98 | 0.94 | 0.96 | 0.95 |  |
| Duration | 275 | 0.92 | 0.79 | 0.85 | 0.85 | 0.81 | 0.83 |  |
| Primary Caregiver (family supporter) Relation | 1460 | 0.97 | 0.97 | 0.97 | 0.98 | 0.86 | 0.92 |  |
| Status Change | 264 | 0.99 | 0.96 | 0.98 | 0.94 | 0.86 | 0.92 |  |
