# Supplementary material for: Real-World Insights Into Dementia Diagnosis Trajectory and Clinical Practice Patterns Unveiled by Natural Language Processing: Development and Usability Study
Source: JMIR Aging. 2025 Feb 25;8:e65221. doi: 10.2196/65221 (PMC11878476; doi:10.2196/65221)
Supplement: Multimedia Appendix 4 [file aging-v8-e65221-s004.docx]

**Multimedia Appendix 4.** Descriptive statistics of providers and insurance information.

| **Total** | **581 (100%)** |
| --- | --- |
| **The location of the first memory loss complaint** |  |
| Geriatrics | 308 (53%) |
| Neurology | 39 (6.7%) |
| Primary care | 185 (31.8%) |
| other | 49 (8.4%) |
| **The location of the first diagnosis of dementia** |  |
| Geriatrics | 350 (61.0%) |
| Neurology | 61 (10.6%) |
| Primary care | 163 (27.4%) |
| other | 7 (1%) |
| **Primary Insurance** |  |
| Medicaid | 72 (12.4%) |
| Medicare | 354 (60.9%) |
| Commercial | 152 (26.2%) |
| No insurance | 2 (0.3%) |
