# Supplementary material for: Real-World Insights Into Dementia Diagnosis Trajectory and Clinical Practice Patterns Unveiled by Natural Language Processing: Development and Usability Study
Source: JMIR Aging. 2025 Feb 25;8:e65221. doi: 10.2196/65221 (PMC11878476; doi:10.2196/65221)
Supplement: Multimedia Appendix 5 [file aging-v8-e65221-s005.docx]

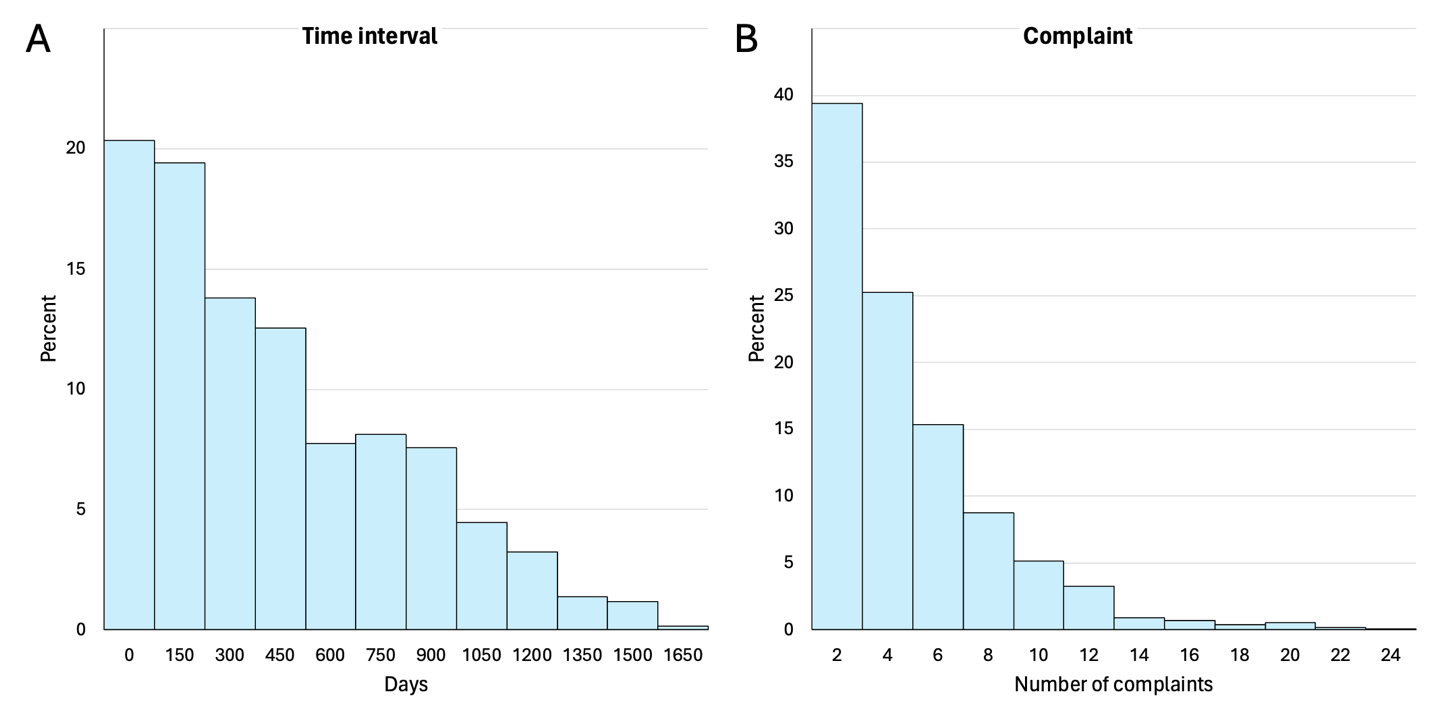


Supplemental Figure 1. The distribution time intervals and complaints. A) Distribution of the time intervals between the first memory loss complaints and the diagnosis of dementia B) Distribution of the number of complaints made before the diagnosis of dementia
